# Modular microfluidic system for on-chip extraction, preconcentration and detection of the cytokine biomarker IL-6 in biofluid

**DOI:** 10.1038/s41598-022-13304-z

**Published:** 2022-06-08

**Authors:** Lucile Alexandre, Amel Bendali, Iago Pereiro, Madad Azimani, Simon Dumas, Laurent Malaquin, Thanh Duc Mai, Stéphanie Descroix

**Affiliations:** 1 Laboratoire Physico Chimie Curie, Institut Curie, PSL Research University, CNRS UMR168 Paris, France; 2 Institut Pierre-Gilles de Gennes, Paris, France; 3 LAAS-CNRS, Université de Toulouse, CNRS, INSA, 31400 Toulouse, France; 4 Université Paris-Saclay, CNRS, Institut Galien Paris-Saclay, 92296 Châtenay-Malabry, France

**Keywords:** Cytokines, Analytical chemistry, Chemical biology

## Abstract

The cytokine interleukin 6 (IL-6) is involved in the pathogenesis of different inflammatory diseases, including cancer, and its monitoring could help diagnosis, prognosis of relapse-free survival and recurrence. Here, we report an innovative microfluidic approach that uses the fluidization of magnetic beads to specifically extract, preconcentrate and fluorescently detect IL-6 directly on-chip. We assess how the physical properties of the beads can be tuned to improve assay performance by enhancing mass transport, reduce non-specific binding and multiply the detection signal threefold by transitioning between packed and fluidization states. With the integration of a full ELISA protocol in a single microfluidic chamber, we show a twofold reduction in LOD compared to conventional methods along with a large dynamic range (10 pg/mL to 2 ng/mL). We additionally demonstrate its application to IL-6 detection in undiluted serum samples.

## Introduction

Biomarkers are considered as objective quantizers of biological processes and particularly pathophysiological processes; they can be used for patient diagnosis or prognosis as well as to monitor disease progression or patient response to treatment. Biomarkers provide guidance through the development of new medicines^1–3^ and are pivotal to decipher molecular or cellular mechanisms involved in pathologies. The increased interest for biomarkers has been accompanied by the emergence of a wide range of bioanalytical developments such as mass spectrometry or high throughput screening.

Among the different biomarkers (cellular, molecular, vesicular), proteins have significantly demonstrated their potential and many of them are analysed and quantified for clinical diagnosis of diseases from asthma and allergies^4,5^ through infections^6^ and cancer^7^. Cytokines are small proteins involved in cell signalling often used as indicators for disease monitoring^8^ such as in tumor progression^9^, liver diseases^10,11^ or hepatic inflammations and fibrosis^12^. In particular, interleukin 6 (IL-6) is involved in the response of the human immune system to infection and cellular injury^13,14^, being secreted by T cells and macrophages into the serum in case of acute and chronic inflammation. Recently, it has been suggested that coronaviruses may activate dysregulated host immune responses. Exploratory studies have suggested that interleukin-6 (IL-6) levels are elevated in cases of complicated COVID-19^15,16^. Thus, a quantitative analysis of cytokines in bodily fluids, and IL-6 in particular, can benefit the monitoring of a wide range of diseases. The current standard methods to detect and analyse cytokines are immunoassays, typically in the form of ELISA, microarrays and bead-based assays^17^. While immunoassays can be highly specific and sensitive, cytokine detection by batchwise immunoassay remains challenging due to their very low concentrations in biological samples down to sub pico or femto—molar concentration^18–20^.

The potential benefits of microfluidics are multi-fold: decrease analysis time, improve bioassays sensitivity, reduce sample and reagent volumes, decrease costs and miniaturize and integrate complex protocols. Impressive results of IL-6 detection in microfluidic systems have already been published relying on glass capillary^21^, modified controlled-pore glass packet^22^ or carbon nanotube forests^23^. But while several microfluidic systems have already shown their efficiency for cytokine detection and quantification, there is still a need for new technologies and methods that can tackle the challenge of precise detection in complex matrices at low cost.

Here we approach this challenge by improving current immunoassay-based protocols in an integrated bead-based microfluidic format. Immunoassays have been widely implemented in microfluidic^24–27^, initially as a miniaturization of conventional microtiter plate ELISA, the antibody being grafted at the surface of the microchannel^28,29^. To further improve the specific surface of interaction and consequently the surface to volume ratio, solid supports have been inserted in microdevices starting with mechanical trapping of micrometric polystyrene beads functionalized with antibodies^30^. The interest of using microbeads as solid support was exemplified by Teste et al. demonstrating theoretically and experimentally that the kinetics of target analyte capture is improved by using micro- and nano- magnetic particles compared to standard microtiter plates^31,32^. Since then, other strategies have been investigated leveraging electrokinetic and magnetic forces^33–35^, in particular in the form of droplet immunoassays combined with magnetics beads^36–39^. Previously, we developed the microfluidic magnetic fluidized bed, a beads-based microfluidic technology based on a homogeneous suspension of magnetic beads inside a microfluidic chamber^40^. A balance of drag and magnetic forces on the beads results in physical properties similar to those of a macroscale fluidized bed. The resulting high surface to volume ratio, constant mixing and compatibility with commercial and functionable beads make it attractive for bioanalysis integration. The porosity of the bed of beads plays a key role in the efficiency of the system, as it could affect the sample residence time and diffusion distances to the solid phase. This was demonstrated in a wide range of applications: bacteria analysis in raw samples^41,42^, detection of histone modifications^43^ and as a miniaturized platform for extra-corporeal circulation^44^. However, sensitive protein detection requires relatively complex multi-step protocols, challenging to integrate in a single device.

Here we leverage the fluidized bed as a tool able to perform all the steps of an ELISA protocol for IL-6 detection: specific extraction, preconcentration, enzymatic binding and detection in a single microfluidic chip. We optimize and evaluate the performance of each step and study how the unique physical features of the mobile solid phase can be tuned to improve assay performance. We do this by adjusting the porosity of the system and the arrangement of the beads according to the molecular diffusion constant and the characteristics of the enzymatic reaction. Finally, we compare the results of our optimized system to the performance of current standard protocols for IL-6 quantitation.

## Materials and methods

### Reagents and chemicals

The washing buffer was prepared with Tris HCl (200 mM, Sigma Aldrich), Bovine Serum Albumin “BSA” (1%, Sigma Aldrich) and Tween 20 (0.1%, Sigma Aldrich). The pH was adjusted at 7.5. The washing buffer was stored at − 4 °C.

The enzymatic substrate MUP (Methylumbelliferyl phosphate, Thermofisher Scientific) was dissolved in the washing buffer at 10 mM, pH adjusted at 8.0. The substrate was kept at − 20 °C.

Tosylactivated beads (DynabeadsTM M-280 Tosylactivated, Thermofisher Scientific) were grafted following the Dynabeads datasheet with anti-human IL6 antibodies (Thermofisher Scientific from the kit CHC1263): they were shaken and incubated with anti-IL6 antibodies, Tris buffer and ammonium sulfate (3 M) at 37 °C for 18 h. Then, the beads were washed and resuspended in PBS with BSA 0.1% at a final concentration of 5 mg/mL. The detection antibodies and enzyme were provided by this same kit.

### Chip fabrication

The microfluidic chip was described in previous publications^40,45^. It consists of an elbow channel leading to a diamond-shape chamber, with an opening angle of 13°. The height of the chamber and channel was set at 50 µm, and the total volume of the PDMS chamber was 0.6 µL.

Microfluidic chips were designed using a micro-milled mold. These molds were machined in brass pieces of 5 cm × 5 cm. The designs were a positive replica of the chip. The chips were fabricated by pouring polydimethylsiloxane (PDMS, Sylgard184, Dow Corning) into the molds (concentration 1:10) and were bonded by oxygen plasma. A surface treatment of PDMA-AGE 0.5%^46^ was incubated inside the chip chamber for 2 h then rinsed with distilled water and dried with compressed air.

### Microfluidic setup

The liquid flow was produced by a pressurization of the sample reservoir using a pressure controller (MFCSTM, Fluigent) allowing to reach a range of pressures from 0.1 mbar to 1 bar, translated in flowrates between 0.1 and 3 µL/min. The outlet of the chip was connected to a flowrate controller (Flowunit S, Fluigent), which allowed precise flowrate measurements and feedback control on the pressure based on the Maesflo software (Fluigent). Peek tubing (Tube Peek 1/32" × 0.25 mm, Cil Cluzeau Info Labo) was used to connect the microfluidic chip to the other elements of the experimental set up. A tube (12 cm peek tubing of 0.063 mm diameter) was positioned at the entrance of the chip to increase the hydrodynamic resistance of the device. A permanent magnet made of NdFeB 1. 47 T (N52, size 20 mm × 20 mm × 30 mm, magnetization direction through the thickness, by ChenYang technologies) was aligned with the chamber axis at a 1.5 mm distance from the chip inlet.

### Operating conditions

#### Manual in-tube labelling ELISA

The sample (50 µL) and the detection antibodies (50 µL, 0.5 µg/mL) were incubated off-chip at room temperature for 50 min with continuous shaking. This mix (50 µL) was flowed inside the chip chamber containing functionalized magnetic beads at a 1 µL/min flowrate. The beads were then rinsed with the washing buffer for 30 min at 2 µL/min inside the chip prior to fluorescent detection.

#### Sequential injection ELISA

The sample (50 µL) was flown inside the chip chamber containing functionalized magnetic beads at 1 µL/min, then the detection antibodies (50 µL, 0.5 µg/mL) were flown at 1 µL/min. The beads were rinsed with the washing buffer for 30 min at 2 µL/min within the chip prior to fluorescent detection.

### Detection

The florescent signal was acquired on the outlet channel of the chamber, at half-height. For both methods, the signal of fluorescence was recorded for 6 min to reach a stable signal and the autofluorescence of the PDMS was subtracted to the signal.

*“Stop-and-go” reading process:* The chamber was filled with 0.7 µL of MUP substrate at 10 mM at a flowrate of 400 nL/min. This step was followed by a 10-min incubation. The fluorescence signal was acquired while 6 µL of washing buffer (Tris HCl 200 mM, 1% BSA, 0.1% Tween 20) was flown through the beads at 1 µL/min.

*In-flow reading process:* The signal was acquired as 6 µL of MUP at 10 mM was flown through the beads bed at 1 µL/min.

### In batch static operating conditions

The experiment in batch was performed in a 1.5 mL Eppendorf tube in which 25 µL of beads were introduced and washed three times in buffer. Beads were incubated for 100 min with the sample (10 ng/mL) or the buffer at room temperature (RT) under stirring, then washed three times. A second incubation was performed with the complex containing the detection antibody and enzyme for 100 min at RT under stirring followed by three washes. MUP substrate at 10 mM (6 µL) was added and incubated for 10 min at RT under stirring. The supernatant is then flown through the microfluidic chip to read the signal.

### Conditions for the calibration curve

It was performed with different concentrations of IL-6 cytokine (Thermofisher Scientific from the kit CHC1263) diluted in PBS and FBS to a final volume of 50 μL.

### Analysis of the data

The analytical characterization of the system was performed with n ≥ 2 and the optimization and calibration curves were obtained with up to 4 replicates for each value. The data were processed through Excel, as well as linear fitting and calculation of correlation coefficient. For Table 1 and Fig. 2, the mean ± Sd is represented. The graphics were design through GraphPad Prism® v5.

## Results and discussion

### Microfluidic and magnetic fluidized bed

Microfluidic magnetic fluidized beds are based on the control of a homogenous suspension of magnetic beads in a microfluidic chamber. The drag force applied on the beads is due to the flow of liquid in the microchannel and is balanced by a magnetic force created by a permanent magnet^40^. Beads are in free suspension in the liquid phase, a configuration that allows to avoid clogging issues. The microfluidic magnetic fluidized bed has shown interesting features regarding biomarkers analysis as it has already demonstrated its efficiency for nucleic acid analysis^47,48^, but so far it has not been applied to protein biomarker analysis in real samples. Here, we put forward the potential of this technology by combining both preconcentration in a dynamic configuration and on-beads detection. First, a high throughput extraction and preconcentration steps of the analyte are performed directly on the magnetic beads, then the detection of the target biomarker by sandwich ELISA is performed on chip and on beads in a very small volume with properties that can be tuned using the fluidized bed properties to achieve optimal performances.

### Off-chip optimization of bead-based assay

#### Bead grafting optimization

To implement a microfluidic fluidized bed-based bioassay for IL-6 detection, we first optimized the bead grafting with capture antibodies for IL-6 capture and preconcentration. In this approach, the magnetic beads in the fluidized bed have a pivotal role as they ensure an efficient extraction of the target in a continuous flow while being used in the second step as solid support to perform the sandwich ELISA. In order to optimize the bioassay performance, we first compared two widely used grafting strategies, based on covalently-grafted capture antibody using either tosyl-activated surface or carboxylic acid modified surfaces (tosyl-activated Dynabeads® and MyOne Carboxylic M-270, respectively)^45^. These grafting strategies have been first compared in tube, using 20 µg of capture antibody per mg of beads for the tosyl-activated beads, and 4 µg of antibody per mg of beads for the MyOne Carboxylic beads as advised by the supplier, the capture antibody being here an anti-human IL6 antibody. To compare the different bead-grafting strategies, sandwich immunoassays were performed in the presence or absence of IL-6 to evaluate the specific to non-specific signal. Data shown in Table S1 demonstrated that the tosyl-activated Dynabeads® allow achieving the highest signal to noise ratio with both positive signal higher and negative signal lower than with carboxylic beads. The tosyl-activated Dynabeads™ as solid phase were thus selected as solid support for the IL-6 bioassay development.

#### Buffer optimization

Further optimizations were performed in tube conditions to determine the best parameters to be integrated on chip. In particular, the choice of washing buffer is very important to limit non-specific adsorption; the performances have been evaluated regarding the immunoassay specificity with different buffer compositions. To do so, washing buffers, with pH ranging from 7 to 8 with different ionic strength and ions/counter ions nature were evaluated: NaHCO_3_ (pH = 8, 100 mM), Tris EDTA (pH = 8, 100 mM), Tris HCl (pH = 8, 200 mM), and PBS (pH = 7, 150 mM). Our studies demonstrate a superiority of Tris–HCl buffer over the other ones (data not shown). In parallel, we have also investigated how the presence of BSA and Tween-20 affects the assay performances, both being well known to limit non-specific adsorption on beads as well as on PDMS. The presence of both additives indeed greatly improves the assay performances (Table S2). Finally, we selected as washing buffer a Tris HCl buffer at 200 mM containing BSA and Tween 20 at a final concentration of 1% and 0.1% (w/v) respectively.

#### ELISA format comparison

Bead-based ELISA is commonly performed by sequential steps including sample incubation, washing, addition of reagent (such as secondary antibody, enzymatic substrate) and magnetic bead separation. But it has been recently demonstrated that single-step immunoassays can reach similar performances. In particular, Mai et al. reported a novel magnetic bead-based immunoassay in which the capture antibodies grafted onto magnetic beads and the detection antibodies can simultaneously bind to Aβ peptides in a single step^45^. We have thus compared both approaches in the microfluidic fluidized bed. In particular, we have evaluated if the formation of the target-detection antibody complex before its injection on chip could affect its extraction and consequently the bioassay performance. We investigated if the formation of the target-detection antibody complex prior on-chip injection could decrease the diffusion constant of the complex and potentially limits its capture in the continuous flow extraction within the fluidized bed.

For the off-chip immune complex formation, the sample, detection antibody and the enzyme were incubated together prior to their on-chip injection (Figure S1.I). The enzyme (Alkaline Phosphatase) was conjugated with the detection antibody through a streptavidin/biotin binding simultaneously with the complex IL-6/detection antibody formation in solution. Magnetic beads were functionalized as previously described (Figure S1.II). The immuno-complex was then injected in the fluidized bed to be captured on the magnetic beads (Figure S1.III). After a washing step to remove the excess of detection antibody and enzyme, the enzymatic substrate was injected within the bed to perform the detection step (Figure S1.IV).

**Figure 1:**
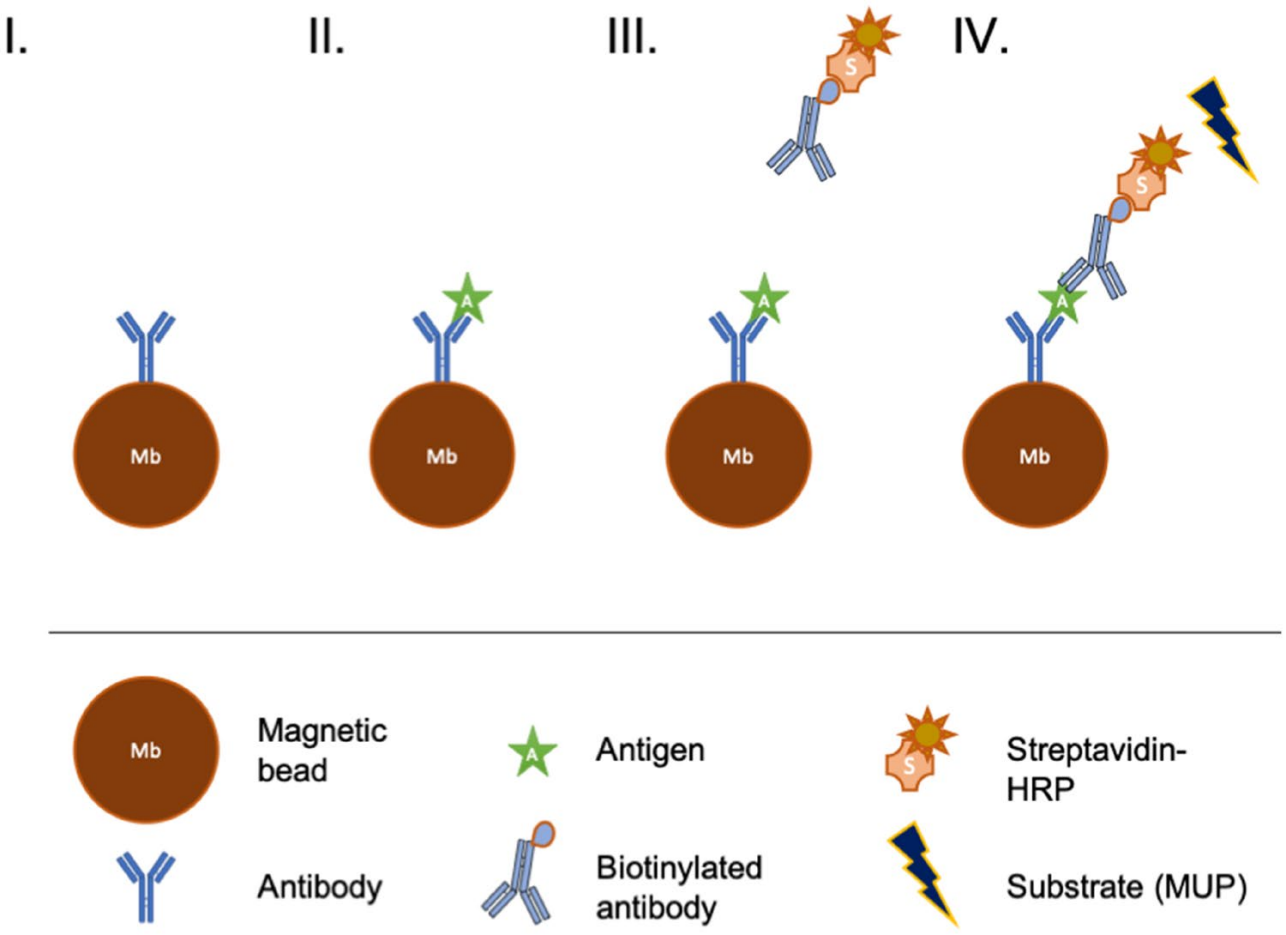
Schematics of the sequential protocol of immuno-capture of the antigen IL-6. (I) the beads coated with the capture antibody are inserted inside the fluidized bed (II) the antigen is flowed through the microfluidic chip and captured on the bead surface (III) the biotinylated antibody is mixed off-chip with the enzyme-streptavidin complex then flowed through the chip and attached to the antigen (IV) the antibody-enzyme detection complex is flowed through the fluidized bed and captured on the beads. Finally, the detection is performed using an enzymatic substrate (MUP).

In the case of the sequential injection, only the conjugation of the detection antibody by the enzyme was performed in tube prior to on-chip injection. Magnetic beads were functionalized as previously described (Fig. 1I). Then, as in a conventional ELISA in microtiter plate, each step was performed sequentially by injecting in the fluidized bed of each solution as follows: the sample was first injected within the fluidized bed (Fig. 1II), a washing step was performed, conjugated detection antibody was injected inside the chip (Fig. 1III), a washing step was repeated, and then the enzymatic substrate was finally injected to perform the detection (Fig. 1IV).

A series of on-chip experiments were conducted to compare these two approaches. Our results showed that a first pre-incubation seemed to slightly enhance the raw signal of detection of the antibody-enzyme complex. These results are in good agreement with previous studies^49,50^. In contrary, the nonspecific signal was significantly lower for sequential injection and the signal to noise ratio was two times higher for sequential injection compared to manual in-tube labelling (Table 1). The fluidization and continuous injection through the suspension of beads allows to reduce non-specific binding to the magnetic beads compared to in tube incubation. As our final goal is to inject complex matrices such as serum within the fluidized bed, the reduction of non-specific interaction needs to be prioritized. In addition, the sequential injection had the advantages of simplified automation. Thus, we selected the sequential injection mode for all subsequent experiments, for which the microfluidic fluidized bed features can be optimized to reach lower limits of detection.

**Table 1:** Influence of the process of injection on the specific and non-specific signal. The experiments are performed with a sample of IL-6 at 10 ng/mL (for the specific signal) or a buffer solution mL (for the non-specific signal) as described in the Material and Methods.

Mode of injection	Mean raw specific signal (u.a.)	Mean raw non specific signal (u.a.)	Signal to noise ratio (u.a.)
Manual in-tube labelling	1821 ± 334	425 ± 51	4.2
Sequential injection	1634 ± 59	201 ± 31	8.1

#### Optimization of the detection step

**Figure 2:**
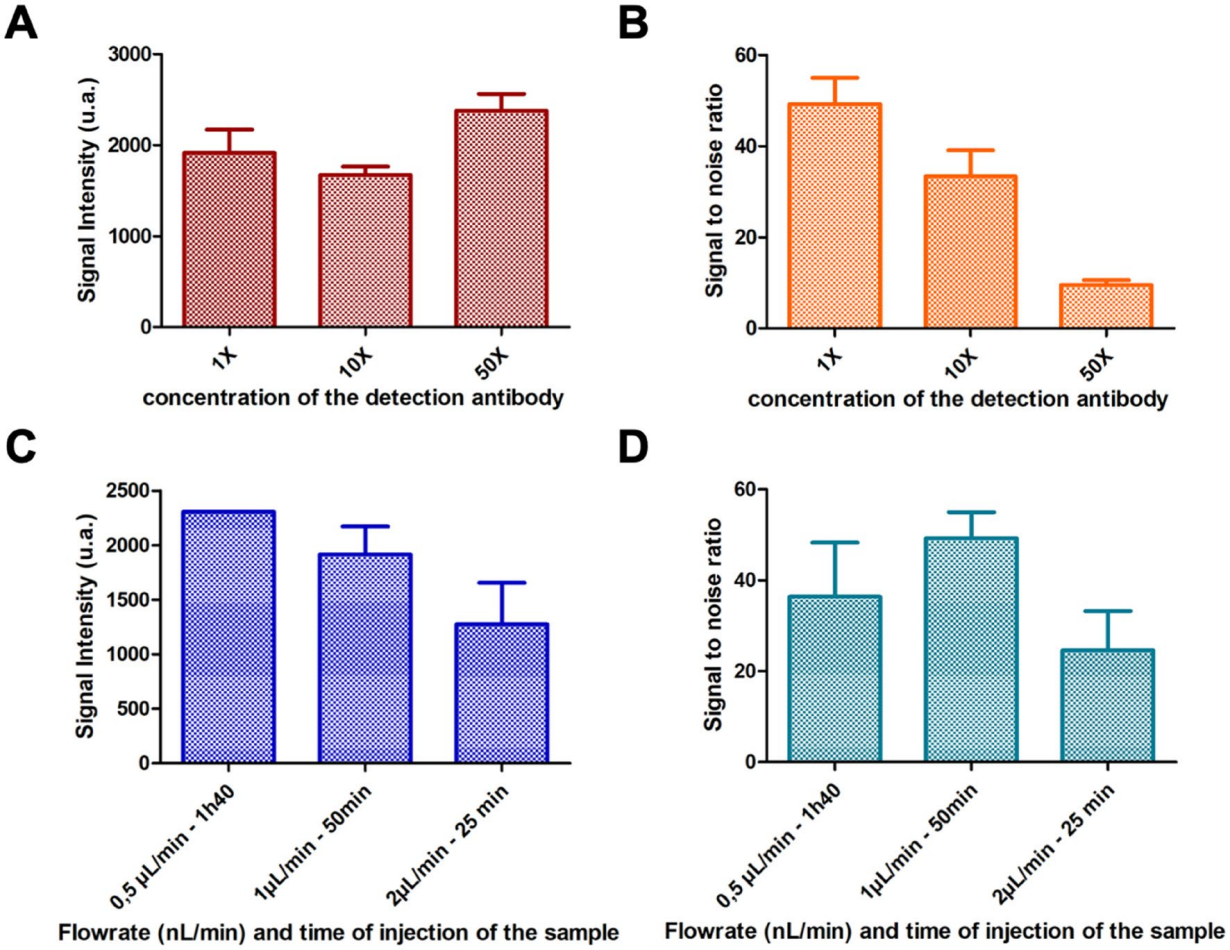
On-chip assay optimization. Signal intensity for a 5 ng/mL sample (**A**) and signal to noise ratio (**B**) of the performed manually in-tube labeling ELISA mode as a function of the concentration of the detection antibody: 0.5 µg/mL, 5 µg/mL and 25 µg/mL. Signal intensity for a 5 ng/mL sample (**C**) and signal to noise ratio (**D**) of the immunoassay as function of the flowrate and the time of injection of the sample for three conditions: 0.5 μL/min, 1 μL/min and 2 μL/min. The analysis was performed as described in the Material and Methods.

The immunoassay format being selected, the detection antibody concentration was next optimized as a compromise between sensitivity and specificity. As previously described, the detection antibody used is an anti-human IL-6 biotinylated antibody conjugated with a streptavidin Alkaline Phosphatase enzyme. The 1X concentration corresponds to a detection antibody concentration of 0.5 µg/mL. The concentration of the antibody-enzyme complex was thus varied between 0.5 µg/mL (1 X) and 25 µg/mL (50 X). As shown in Fig. 2A, the intensity of the signal obtained in the presence of IL-6 at 5 ng/mL can be slightly increased when increasing the detection antibody concentration. However, this goes hand in hand with a significant increase of the non-specific signal and a decrease of the signal to noise ratio (Fig. 2B). The optimal concentration of detection antibody was set at 0.5 µg/mL, condition for which the higher signal to noise ratio was reached while reducing the cost per assay.

### On-chip assay optimization and influence of fluidization parameters

With the conventional parameters of the bead-based immunoassay optimized, we next leveraged the fluidized bed format to improve assay performance. The microfluidic magnetic fluidized bed design has been optimized to reach a high homogeneity of bead distribution within the microchamber^40^, but it has also been shown that the bead bed porosity can be tuned at will within the chip. A change of flowrate induces a change of the drag force applied to the beads. The balance between drag and magnetic forces is modified so that bed of beads expands as the flowrate increases. The influence of the flow rate on the on-chip immunoassay has thus been investigated as a change in porosity can impact not only the analysis time (from 25 to 100 min for 50 μL of sample) but also the diffusion distance, as well as the residence time of the target biomolecule within the bed of particles.

From previous work^40^, we know that the magnetic beads in microfluidic fluidized beds tend to self-organize in cylindrical clusters of diameter d_c_ ≈12 μm due to bead-bead interactions. In a simple 1D approximation, we can consider the bed porosity ε being defined by the distance between these clusters d_s_ and their size d_c_:

1 $$ \begin{array}{*{20}c} {\varepsilon = \frac{{{\varvec{d}}_{{\varvec{s}}} }}{{{\varvec{d}}_{{\varvec{c}}} + {\varvec{d}}_{{\varvec{s}}} }}} \\ \end{array} $$

For an efficient capture, the analyte needs to be able to reach, by diffusion, a magnetic bead before leaving the fluidized bed due to the flow-driven convection. If we call t_d_ the time to reach a surface of capture by diffusion and t_c_ the time to cross the magnetic bed by convection, we need to ensure that $\frac{{{\varvec{t}}_{{\varvec{d}}} }}{{{\varvec{t}}_{{\varvec{c}}} }}\user2{ } \ll 1$, so that the antibody-antigen interaction can occur effectively within the residence time.

The time t_d_ needed to travel the distance d = d_s_/2 allowing an analyte to reach the closer cluster by diffusion can be estimated with Einstein’s relation:$\user2{ t}_{{\varvec{d}}} = \user2{ }\frac{{{\varvec{d}}_{{\varvec{s}}}^{2} }}{{8{\varvec{D}}}} = \user2{ }\left( {\frac{{\user2{ \varepsilon }}}{{1 - \user2{ \varepsilon }}}} \right)^{2} \frac{{{\varvec{d}}_{{\varvec{c}}}^{2} }}{{8\user2{ D}}}$ , where D is the diffusion constant of the analyte. On the other hand, the residence time of the analyte within the bed can be approximately evaluated as ${\varvec{t}}_{{\varvec{c}}} = \user2{ HL}^{2} \tan \left( {\frac{{\varvec{\alpha}}}{2}} \right)/{\varvec{Q}}$, where L is the bed’s length, α the aperture angle of the chamber and H the chamber height. Hence, the ratio between both times is:

2 $$ \begin{array}{*{20}c} {\frac{{{\varvec{t}}_{{\varvec{d}}} }}{{{\varvec{t}}_{{\varvec{c}}} }} = \left( {\frac{{\user2{ \varepsilon }}}{{1 - \user2{ \varepsilon }}}} \right)^{2} \frac{{{\varvec{d}}_{{\varvec{c}}}^{2} {\varvec{Q}}}}{{8\user2{ L}^{2} {\varvec{HDtan}}\left( {\frac{{\varvec{\alpha}}}{2}} \right)}} \ll 1} \\ \end{array} $$

Considering a diffusion constant of cytokine IL-6 D = 8.5 10^–8^ cm^2^.s^−1^^51^ and the aperture angle of the fluidized bed being α = 35° = 0.61 rad, we can estimate the ratio of times based on published measures of the bed length L and porosity ε^40^ at flowrates of 0.5, 1 and 2 μL/min (Table 2).

**Table 2:** Evolution of the ratio t_d_/t_c_ between the time of diffusion between the capture beads clusters and the residence time inside the fluidized bed due to the flowrate Q of the liquid percolating the bed.

Q [uL/min]	0.5	1	2
$\frac{{{\varvec{t}}_{{\varvec{d}}} }}{{{\varvec{t}}_{{\varvec{c}}} }}$	0.32	0.82	3.07

While this remains an approximation, note that the ratio $\frac{{{\varvec{t}}_{{\varvec{d}}} }}{{{\varvec{t}}_{{\varvec{c}}} }}\user2{ }$ approaches 1 for a flowrate of 1 μL/min, already significantly above 1 for a flowrate of 2 μL/min (Table 2). Hence, we would expect our system to efficiently promote the interactions between the analyte and the surface of capture of the beads up to a maximum flowrate of ~ 1 μL/min.

We thus experimentally investigated how the flowrate of the sample injection impacts the specific signal intensity at the outlet of the chip (Fig. 2C). Our experiments showed that a shorter residence along with larger distance to the particles can significantly affect the immunoassay performances, the fluorescence intensity decreasing as the flowrate increases, in agreement with our model previously described. As a compromise between the assay sensitivity and the analysis time, the flowrate was set at 1 µL/min, allowing to reach the higher signal to noise ratio (Fig. 2D). In those conditions, the signal intensity is decreased by 20% compared to a slower flowrate but the analysis time is divided by two and the background noise is decreased by 40%. Furthermore, the correlation between the volume of the sample and the intensity of the detected signal was investigated and we were able to show a high correlation (Figure S2), showing the versatility of our device towards the volume of sample.

Finally, the choice of the fluidized bed to integrate IL-6 immunoassay was also motivated by its unique modularity to improve the immunoassay performance. As fluidization occurs when passing liquid through a packed bed of particles at a sufficient velocity to compensate magnetic forces, two regimes can be achieved with this device: below a threshold flowrate, the beads are in close contact and organized as a packed bed of particles, while above this flowrate interparticle distance increases resulting in higher porosity and improved fluid/solid contact in a fluidized bed regime. These two regimes (packed bed and fluidized bed) can then coexist in our system as a function of the flow rate applied and have been compared here to improve the last step of the immunoassay: the enzymatic reaction. The enzymatic reaction taking place within the fluidized bed offers the possibility to consider two detection modes with potentially improved performances. Indeed, after injection of the enzymatic substrate in the bed, the fluorescent signal can be detected either continuously (in flow method) or by sequentially changing the flowrate above and below the threshold (stop-and-go method).

**Figure 3:**
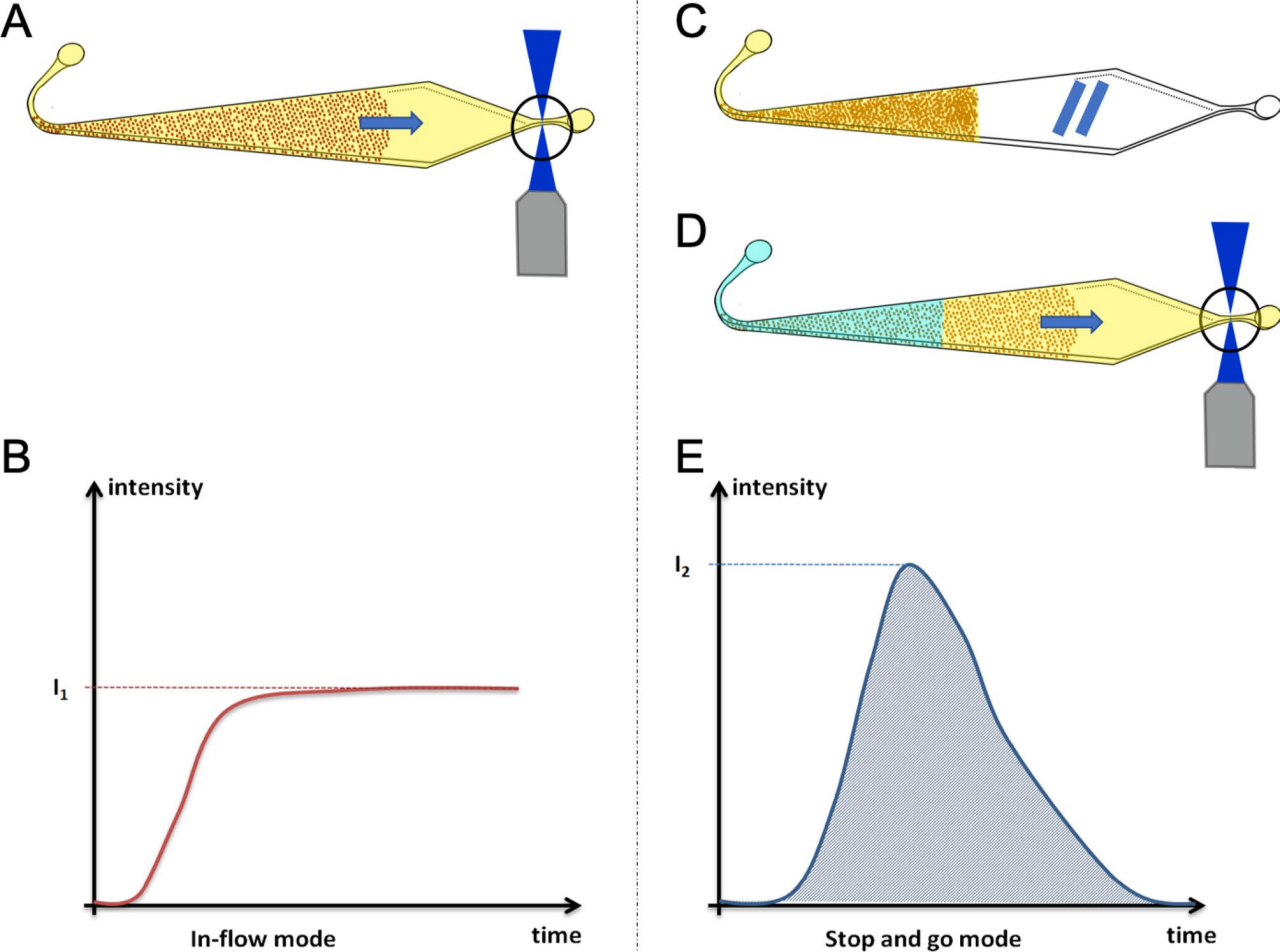
Schematics of the in-flow and 'stop-and-go' detection steps. The panel (**A**) described the in-flow mode where the substrate solution is flown through the beads. The two next panels describe the ‘stop-and-go’ process. The matrix of beads is filled with the substrate solution. An incubation of 10 min at RT is performed (**C**) then the liquid is pushed by the buffer to the exit channel where the fluorescence is measured (**D**). The signals obtained have different shapes for the in-flow mode (**B**) and the stop-and-go mode (**E**).

In the continuous in-flow detection approach, the enzymatic substrate was flowed continuously inside the chamber at 1 µL/min for 6 min (Fig. 3A). The bed is, at this flow rate, in a fluidized bed regime; the enzymatic product generated at the surface of the beads is thus continuously flowed through the bed to reach the detection area. The signal has a shape of an asymptotic curve (Fig. 3B). The value of interest is the height of the plateau, proportional to the concentration of IL-6 in the initial sample.

In the stop-and-go method, a given volume of enzymatic substrate (0.7 µL) is first flowed through the bed at 0.4 µL/min. The pressure is then decreased so that the substrate and the magnetic beads can be incubated in a packed bed regime for 10 min decreasing drastically the diffusion distances between the beads and the enzymatic substrate (Fig. 3C). After 10 min of incubation, the pressure is increased with a retro-controlled program so that the solution is flowed towards the area of detection at 1 µL/min (Fig. 3D). A fluorescence peak is thus obtained (Fig. 3E) as the quantity of product obtained during the 10-min incubation being a finite quantity. The opening of the bed after the 10 min of incubation is a critical step which could affect the shape and dimensions of the peak. By this process, a high quantity of fluorescent product can be accumulated inside the bed of beads before reaching the detection area while switching on the flow rate. With this approach, we aimed at increasing the IL-6 immunoassay sensitivity. The differences in the shapes of the recorded signals are related to the physical properties of the fluidized bed such as the porosity of bead assembly.

As shown in Table 3, the signals of three quantification methods of the fluorescent signal were compared to choose the most accurate one. A higher signal was recorded when working with the stop-and-go mode, as expected. Interestingly, the coefficient of variation was smaller when using the peak area measurement rather than the peak height whereas the mean specific signal was higher. It allowed us to reach a signal to noise ratio almost as high as the one of in-flow mode, but with a mean specific signal more than 3 times higher. Finally, despite quite similar performances in terms of signal to noise ratio, we selected the stop-and-go mode rather than the in-flow one in order to reach higher specific signal to lower the limit of detection. Our analytical model showed that an increase of the flowrate above 1 μL/min would limit the antigen–antibody interaction, not allowing improvements when working with the continuous mode. A solution to circumvent this issue lies in the addition of the incubation steps. This choice left more freedom for further optimization if needed: the sensitivity could be increased by optimizing either the injected volume of enzymatic substrate or the incubation time of the stop-and-go mode.

**Table 3:** Comparison between continuous and ‘stop-and-go’ methods for experiments performed with a sample of IL-6 at 10 ng/mL (for the specific signal) or a buffer solution mL (for the non-specific signal) as described in the Material and Methods.

	In-flow mode	Mean specific signal (u.a.)	Coefficient of variation (%)	Signal to noise ratio (u.a.)
Front height	Signal	2038	11.2	33.3

	**Stop-and-go mode**			
Peak area	Signal	7409	15.9	30.6
Peak height	Signal	2834	21.0	9.4

### Evaluation of the performance of the system for IL-6 detection

**Figure 4:**
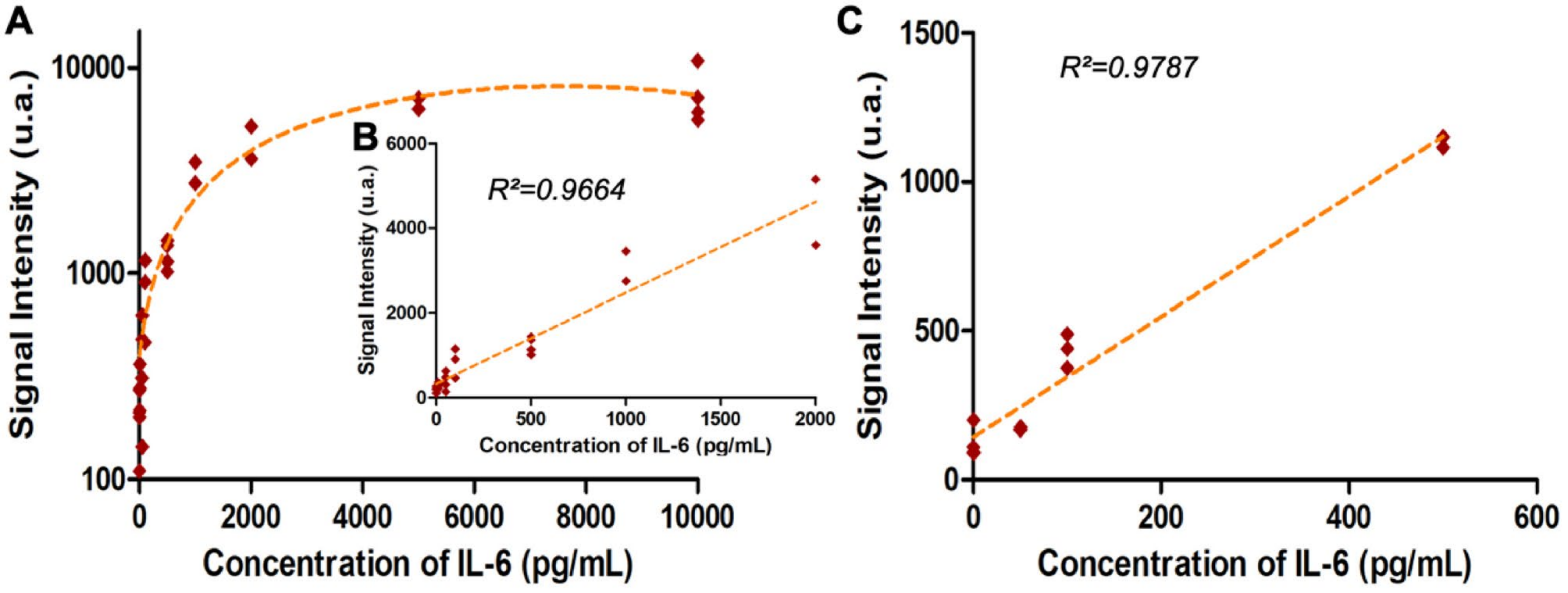
Evaluation of the performance of the system. (**A**) and (**B**) IL-6 Immunoassay calibration curve, on chip signal intensity as function of the IL-6 concentration in Tris–HCl buffer where the limit of detection is 6 pg/mL and C) linear dynamic range of the calibration curve with IL-6 spiked in Fetal Bovine Serum (FBS) (annotation of the coefficient of regression for each linear regression). The analysis was performed as described in the Material and Methods.

To further evaluate the performances of the fluidized bed-based ELISA in terms of dynamic range and sensitivity, we established a calibration curve with the sequential injection protocol and a detection performed by stop-and-go mode for samples of 50 μL. This series of experiments was first performed with a wide range of IL-6 concentrations ranging from 10 pg/mL to 10 ng/mL in Tris HCl buffer 200 mM (at pH 7.5 with BSA at 1% and Tween 20 at 0.1%) as shown on Fig. 4A. The figure plotted in the log–log scale can be find in the supplementary (Figure S3).

A 50 µL sample was flowed through the chip at 1 µL/min then the detection antibody (0.5 µg/mL) was injected at room temperature for 50 min at the same flowrate. A quite large dynamic range was obtained with linear response from 10 pg/mL to 2 ng/mL, as shown in Fig. 4B. This dynamic range of our approach is competitive compared to those reported in the literature^52,53^ or to commercial immunoassays^54^ usually going from few pg/mL to ng/mL. Negative controls were performed with non-spiked buffer. We next evaluated the sensitivity of our newly developed immunoassay on the basis that significant signal is three standard deviation above the negative control. We obtained a limit of detection (LOD) at 6 pg/mL, more than two times lower than the one of the standard beads immunoassay (LOD is 15.6 pg/mL from manufacter’s data).

We finally applied the on-chip method to complex samples and performed a calibration curve with IL-6 spiked in fetal bovine serum (FBS) to validate our integrated approach towards complex sample matrix analysis (Fig. 4C). This was performed on a reduced range of concentration, closer to clinical sample conditions. The results show a strong similarity with the results achieved in Tris–HCl, with a linear response in the whole dynamic range and an LOD of 60 pg/mL. Due to its tunable porosity, the microfluidic magnetic fluidized bed is well suited to work with complex matrices. However, as expected due to the high protein content of serum, it is associated with a slightly decreased sensitivity. We assume that some screening effect due to the high protein content of the serum extraction could affect IL-6 specific capture and consequently the assay sensitivity.

Altogether our results showed that the tunable properties of a magnetic and microfluidic fluidized bed allows to integrate an automated sequence of on-chip extraction and detection of Il-6. The fluidization regime can be used to limit the non-specific interactions and avoid clogging when working with complex matrices whereas the packed state could be used to enhance the detection step. The control of these two regimes (packed and fluidized states) allowed us to reach a relevant limit of detection in the tens of pg/mL, compatible with, for instance, the requirements of IL-6 detection in patient serum in sepsis^55–57^. Based on this first proof of concept, the modulable device may be further adapted for the detection of other cytokines.

## Conclusion

Microfluidic systems have demonstrated their potential to enhance bioassay performance and integration, particularly in the case of immunoassays. However, fully integrated multi-step protocols combining analyte extraction, preconcentration and detection in a single module are still challenging, particularly when addressing high sensitivity and/or compatibility with complex matrices. We believe the microfluidic fluidized bed-based approach presented here provides a new way to tackle high-performance immunoassays in fully automated protocols. Its versatility is based on the addition of new variables: the tunable porosity and arrangement of the magnetic beads. In particular, the extraction conditions can be tuned as a function of the diffusion constant of the analyte, while the enzymatic step can further be modified to improve the assay performances. This new technology is also compatible with a large range of biomarker concentrations as well as with sample volumes ranging from one to a few hundred µL.

We demonstrate here that it allows a fast detection of the cytokine IL-6 with a large dynamic range (10 pg/mL to 2 ng/mL), in less than 2 h, with an LOD in the picomolar range. The sensitivity achieved in this first proof of concept is applicable for instance to severe sepsis infection, where IL-6 levels can go up to a few hundreds of pg/mL in human serum samples. A LOD of 6 pg/mL for IL-6 spiked in buffer solution was achieved. This value is lower than the LOD achieved in experiments performed with the same reagents in on-bench conditions, and better overlaps with clinical ranges^19,56^. Moreover, we demonstrated its compatibility with challenging matrices of high protein content and no dilution while still ensuring a clinically-relevant sensitivity. We believe that this capability to enhance the performance of conventional assays while fully integrating complex sequential protocols make this approach a promising tool for future biomarker detection and quantification applications.

## Supplementary Information

Supplementary Information.

## Data Availability

The datasets generated and analysed during the current study are available from the corresponding author on reasonable request.
