# Supplementary material for: Modular microfluidic system for on-chip extraction, preconcentration and detection of the cytokine biomarker IL-6 in biofluid
Source: Sci Rep. 2022 Jun 8;12:9468. doi: 10.1038/s41598-022-13304-z (PMC9176165; doi:10.1038/s41598-022-13304-z)
Supplement: Supplementary file 1 — Supplementary Information. [file 41598_2022_13304_MOESM1_ESM.docx]

**Supplementary Informations**

**Modular microfluidic system for on-chip extraction, preconcentration and detection of the cytokine biomarker IL-6 in biofluid**

***Table S1***: *Influence of the bead’s functionalization on the specific and non-specific signal of sandwich IL-6 immunoassay. The experiment in batch is performed with a sample of IL-6 at 10 ng/mL (for the specific signal) or a buffer solution mL (for the non-specific signal) as described in the Material and Methods*

| Beads used | Mean specific signal (u.a.) | Mean nonspecific signal (u.a.) |
| --- | --- | --- |
| Tosyl-activated Dynabeads™ | 5797 | 375 |
| MyOne Carboxylic Dynabeads™ | 2279 | 1003 |

***Table S2:*** *Influence of the buffer composition on the nonspecific signal.* *The experiment in batch is performed with a sample of IL-6 at 10 ng/mL (for the specific signal) or a buffer solution mL (for the non-specific signal) as described in the Material and Methods*

| Buffer (% w/v) | Nonspecific signal (u.a.) |
| --- | --- |
| Tris HCl 200 mM pH 8 BSA 1% TWEEN 0.1% | 375 |
| Tris HCl 200 mM pH 8 BSA 0.5% TWEEN 0.1% | 404 |
| Tris HCl 200 mM pH 8 BSA 0.5% TWEEN 0.05% | 706 |
| Tris HCl 200 mM pH 8 BSA 0.1% TWEEN 0.1% | 397 |
| Tris HCl 200 mM pH 8 BSA 0% TWEEN 0% | 537 |

Figure S1: Representation of the manual in-tube protocol of immuno-capture of the antigen IL-6 I) Off-chip the biotinylated antibody is mix with the antigen and the complex enzyme and streptavidin II) the beads coated with the capture antibody are inserted inside the fluidized bed III) then the complex antigen-detection antibody-enzyme is flowed through the microfluidic chip and capture on the surface of the beads IV) the detection is performed using a substrate (MUP)


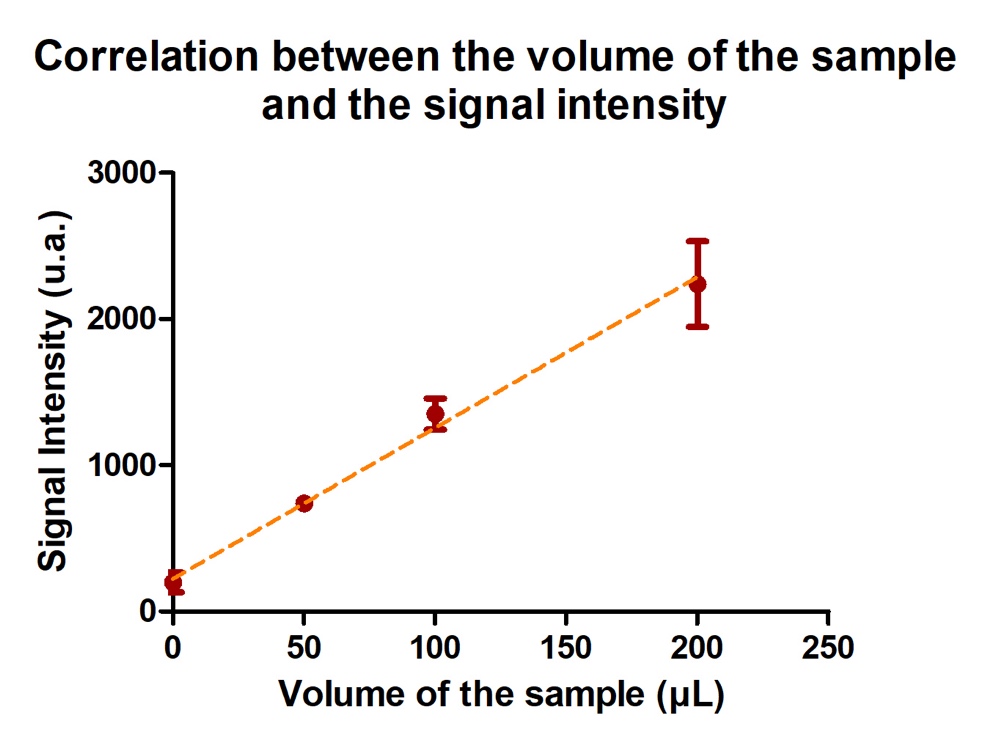


Figure S2: Evaluation of the correlation between the volume of the sample and the intensity of the signal detected. The coefficient of correlation was calculated at r^2^ = 0.9949


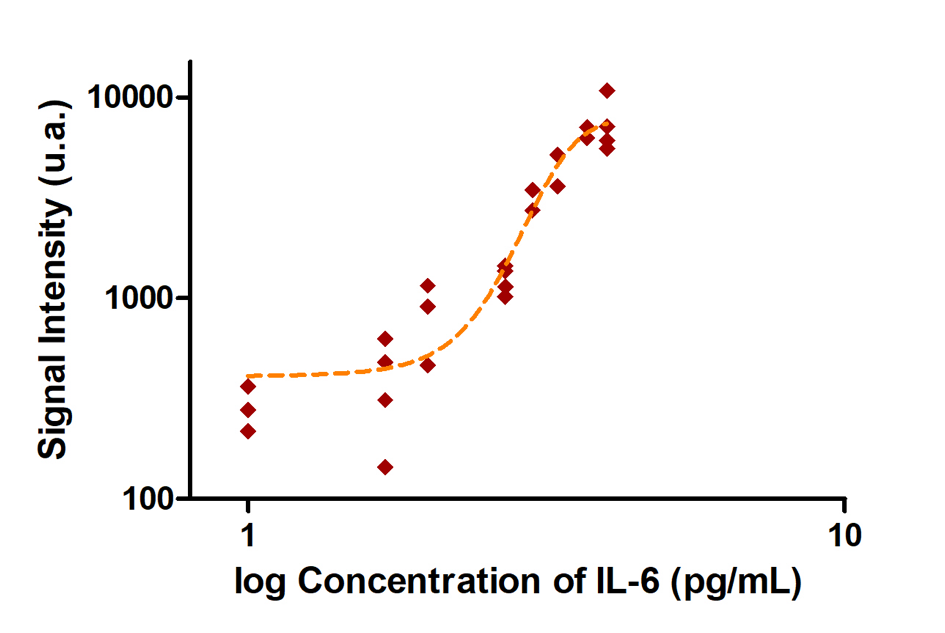


Figure S3: Evaluation of the performance of the system: IL-6 Immunoassay calibration curve, on chip signal intensity as function of the log of IL-6 concentration in Tris-HCl buffer with standard curve. The analysis was performed as described in the Material and Methods
